# The Scientific American Anniversary Number

**Published:** 1896-09

**Authors:** 


### THE SCIENTIFIC AMERICAN. Anniversary number, July 25th, 1896.

The *Scientific American* has reached the mature age of fifty years. It is therefore with commendable pride that its editor and proprietors have prepared a special Anniversary Number, with four times the usual number of pages, to celebrate the occasion. This number contains reviews of the progress made in the last fifty years in the sciences and the arts, gives historical sketches of some of the most notable inventions made during this period, and is filled with interesting illustrations. Among the subjects treated are: The Transatlantic Steamship, Naval and Coast Defense, Railroads and Bridges, the Sewing Machine, Photography, the Phonograph, Telegraph, Telephone, Iron and Steel, Physics and Chemistry, Progress of Printing, the Bicycle, Electric Engineering, Telescopes, Ocean Telegraphy, Distinguished Living Inventors (Portraits), Shipyards of the United States, a large group of distinguished inventors, reproduced from an old steel engraving, is presented. The Anniversary Number is provided with a characteristic cover, and is printed in a style fully up to the regular issues of the paper. It will doubtless be generally preserved for future reference. A very large edition of this interesting number is being issued. All articles have been contributed by specialists, and are of great value as a work of reference. In size, this issue is equivalent to an ordinary sized book of 442 pages. Subscription price, $3 per year, or for the special, 10c. a copy. Munn & Co. Publishers, New York.

